# Injury Patterns in Portuguese Under-23 and Senior Rink Hockey Athletes: A Retrospective Cross-Sectional Study

**DOI:** 10.3390/jfmk11030260

**Published:** 2026-06-29

**Authors:** Sofia Sacadura, Ricardo Maia Ferreira, Maria Paula Pacheco, Rui Soles Gonçalves

**Affiliations:** 1 Scientific-Pedagogical Unit of Physiotherapy, Coimbra Health School, Polytechnic University of Coimbra, 3045-043 Coimbra, Portugal; sofiarochasacadura2@gmail.com (S.S.); rferreira@ipmaia.pt (R.M.F.); paulap@estesc.ipc.pt (M.P.P.); 2 Social Sciences, Education and Sport Department, Polytechnic Institute of Maia, N2i, 4475-690 Maia, Portugal; 3 Sport Physical Activity and Health Research & Innovation Center (SPRINT), 4960-320 Melgaço, Portugal; 4 Health & Technology Research Center (H&TR), Coimbra Health School, Polytechnic University of Coimbra, 3045-043 Coimbra, Portugal

**Keywords:** rink hockey, epidemiology, musculoskeletal injuries, injury prevention, Portugal

## Abstract

**Background:** Rink Hockey is a high-intensity contact sport with growing participation in Portugal, yet epidemiological data on injuries among senior and under-23 practitioners remain scarce. This study aimed to retrospectively describe self-reported injury occurrence, injury characteristics, and potential associations with demographic and sport-related variables among Portuguese Rink Hockey athletes. **Methods:** A retrospective, cross-sectional, self-reported e-survey was conducted among federated Portuguese Rink Hockey practitioners (under-23 and senior categories) during the 2024/2025 season. The questionnaire included 53 closed-ended items on sociodemographics, sport participation, equipment, training loads, and injury history. Injury prevalence, incidence rate, mean injuries per athlete, and associations were analyzed. **Results:** Among 181 respondents (68.5% male; age 22.3 ± 4.3 years; experience 15.8 ± 5.0 years), 89 (49.2%) reported at least one injury (mean 2.6 ± 2.7 injuries/athlete in the total sample; 3.3 ± 3.1 per injured athlete). Estimated incidence was 3.9 ± 5.9 injuries/1000 h (total sample) and 7.9 ± 6.2/1000 h (injured athletes). The knee (19.1%) was the most common injury localization, and muscular injuries (25.8%) were the most frequent type. Most injuries occurred during matches (46.0%), with contact with another player (27.0%) during offensive transition (40.4%) in areas surrounding the goal (57.3%) being the most frequently reported circumstances. Older and female athletes reported a higher injury prevalence than younger and males counterparts (66.7% vs. 33.3% [*p* = 0.042; ES = 0.174] and 61.4% vs. 43.5% [*p* = 0.026; ES = 0.166], respectively). Injury occurrence was positively associated with age (r = 0.262–0.158, *p* ≤ 0.05) and playing experience (r = 0.157, *p* ≤ 0.05). However, greater playing experience was associated with lower odds of joint injury (11–15 years: OR = 0.116, 95% CI [0.019; 0.695], *p* = 0.018; 16–19 years: OR = 0.116, 95% CI [0.019; 0.695], *p* = 0.018; and ≥20 years: OR = 0.056, 95% CI [0.006; 0.534], *p* = 0.012). **Conclusions:** Portuguese Rink Hockey practitioners exhibit a high injury burden, predominantly affecting the knee, with muscle injuries and contact/overuse mechanisms as major contributors. Age, sex, and experience were associated with injury occurrence. These findings may support the incorporation of targeted prevention strategies into multidisciplinary support teams.

## 1. Introduction

Rink Hockey is a team sport that emerged in England at the end of the 19th century and is currently practiced internationally. Among the countries where its sporting relevance is most evident, Portugal, Spain, Italy, and Argentina have not only the largest number of registered athletes but also the highest number of titles won in international competitions [1,2].

The practice of this sport is characterized by the use of specific equipment. In this context, it includes a ball made of pressed rubber/plastic with a cork core, as well as four-wheeled quad roller skates arranged along two transverse axles, including a brake at the front, and a stick consisting of a shaft and a curved end, called a blade, made of wood and/or fiber, used to move the ball. The mandatory equipment for outfield players includes a jersey or T-shirt, shorts, socks, two boots with skates and a stick, while goalkeepers use specific protective equipment [3].

The game is played on a rectangular rink, which can be covered or outdoors, with dimensions that can vary between 17 and 22 m in width and between 34 and 44 m in length; the four corners are rounded. The main objective is to put the ball into the opponent’s goal while preventing the opponent from scoring. Rink Hockey is a collective sport, where each team can have up to ten players, two of whom must be goalkeepers, and the minimum number to start a game is three outfield players and two goalkeepers. In senior and under-23 categories, the total duration of a match is 50 min, divided into two halves of 25 min, separated by a 10 min interval [3].

These sport regulatory and spatial characteristics directly condition the demands placed on athletes during practice [4]. From the perspective of game dynamics, Rink Hockey is distinguished by a high execution speed and frequent situations of physical contact. The ball can reach speeds close to 160 km/h and players around 70 km/h, factors that increase the risk of injury and potential compromises in athlete performance and health [5]. Physiologically, this dynamic translates into a succession of high-intensity efforts, placing Rink Hockey among intermittent sports. In this context, athletes perform short duration, high-intensity actions such as sprints, accelerations, and decelerations, interspersed with periods of lower physical demand, which imposes a high load on the cardiovascular and metabolic systems. During matches, athletes present heart rate values ranging between 85 and 95% of maximum heart rate, while blood lactate concentrations average 4.5–7.2 mmol·L^−1^, reflecting a significant contribution of glycolytic anaerobic metabolism [6]. In addition to these acute responses, aerobic capacity is a key determinant of recovery between high-intensity efforts and sustained performance. It is expected that during matches, athletes spend ~40% of the time in the light intensity zones (<70% of HRmax), ~15% in the aerobic zone (70–80% HRmax), ~24% within the anaerobic zone (80–90% HRmax), and ~17–20% into the maximum effort zone (>90% HRmax) [7]. Although few studies have directly evaluated maximum oxygen consumption in elite male players, data from laboratory tests and simulation protocols report values between 50 and 60 mL·kg^−1^·min^−1^ [8]. Rink Hockey also requires the integrated development of endurance, strength, power, speed, and agility [8,9,10].

However, these same physical and physiological demands that sustain competitive performance can, when associated with high training and competition volumes and intensities, simultaneously contribute to an increased risk of injury. The intermittent high-intensity nature of Rink Hockey, with repeated sprints and abrupt changes of direction, imposes significant mechanical and metabolic loads that may increase susceptibility to musculoskeletal injuries. Therefore, the analysis of sports injury epidemiology becomes particularly relevant, as it allows us to understand the impact of these demands on injury risk through population-level injury analysis and the identification of intrinsic and extrinsic risk factors. This knowledge is fundamental for the development of effective prevention and treatment strategies within Rink Hockey [11].

Despite growing scientific interest, the epidemiological literature on Rink Hockey remains relatively scarce. Studies demonstrate a consistent injury incidence in Rink Hockey, with values ranging between 3.23 and 14.2 injuries/1000 h, being systematically higher in game contexts (matches: ~45–75%, 17.5–36.7 injuries/1000 h; training: ~25–55%, 1.77–7.5 injuries/1000 h). In men’s, the incidence can reach 9.8 injuries/1000 h, whereas in female athletes, reach 8.9 injuries/1000 h. Of these injuries, overuse (13.6–45.8%) and contact injuries (22.7–35.4%) are the most frequent reported mechanisms. Regarding the type and location of injuries, there is a predominance of joint (11.3–44.4%), muscle/tendon (25–43.7%) and bone (3.7–22.7%) injuries, with particular incidence in the lower limbs (34.1–65.4%), followed by the upper limbs (27.1–31.8%), head/neck (1.2–20.8%), and trunk/back (2.5%). Concerning injury severity, the median time loss was 0.9 ± 1.4 weeks per player per season and 12.7 ± 20.3 weeks per player career, with ankle/feet (13.7 ± 24.2 days), knee (11.1 ± 24.3 days), and groin/hip injuries (10.1 ± 29.9 days) being the most severe [1].

With the methodological diversity of the studies presented, they allow the identification of trends regarding injury incidence and patterns in Rink Hockey. However, the heterogeneity of study designs and competitive contexts limit the direct comparison and generalization of results. At the same time, the scientific literature shows that higher levels of adherence to injury prevention programs are associated with reduced injury rates, while lower levels of adherence do not result in a significant decrease in injury incidence. Prevention programs can reduce injury risk in different personal and sports contexts. In this sense, understanding injury patterns observed in the sport is a fundamental step for the development and implementation of effective prevention strategies adapted to the context, highlighting the need for additional research with more standardized methodologies and targeted at specific populations and competitive contexts [12].

Although some competitive contexts have been partially characterized, and despite the widespread practice and importance of Rink Hockey in Portugal, research on injury epidemiology within its competitive context remains limited, particularly at the national level. Given the importance of the under-23 category as a transitional stage to senior competition, and the frequent overlap between under-23 and senior participation, this study examined injuries in under-23 and senior athletes from both female and male first-division competitions during the 2024/2025 season in Portugal. Specifically, the aims of the study are to describe injury prevalence and incidence, identify the most common injury types and affected body regions, characterize time loss from sport participation, and explore potential factors associated with injury occurrence, including athlete characteristics, training and competition load, and practice context.

## 2. Materials and Methods

To achieve the objectives, a retrospective, cross-sectional, self-reported study was conducted, following established methodological guidelines [13,14,15,16]. This study followed the Ethical Principles of the Helsinki Declaration [17] and was approved by the Polytechnic Institute of Coimbra Ethics Committee. Before initiating the e-survey (on the front page), the purpose and context of the study, data usage and protection rights, eligibility criteria, instructions about how to fill and complete the e-survey, and the contact for clarifications were explicitly stated. The consent for participation in the study was obtained through an informed consent statement. After agreeing to participate in the study and with the informed consent, the Rink Hockey practitioners were granted access to the e-survey.

### 2.1. Participants and Data Collection

In an attempt to ensure the correct population sample, the potential participants were reached through the various Rink Hockey clubs’ communication channels. At this time, the study’s procedures, objectives, and eligibility criteria were explained. The eligibility criteria for participation in the study were: ability to read and write in Portuguese; being over 18 years old; practicing Rink Hockey; being a federated athlete in the 2024/2025 season; signing the informed consent; completing the questionnaire; self-reported absence of any condition that prevents understanding and completing the questionnaire (e.g., motor, visual, or intellectual disability); residing in Portugal (mainland or islands); of any sex or Rink Hockey experience. The sample size goal was set at 254 responses, based on 744 Portuguese Rink Hockey affiliates in the 2024/2025 season (under-23 and seniors’ men and women, provided by the national Rink Hockey federation), with a 95% confidence level, a margin of error of 5%, and a response distribution of 50% [18]. To ensure that the sample size goal was achieved, a thank you note and a reminder containing the questionnaire link were sent at the two-, four-, and six-week intervals.

### 2.2. Questionnaire

The questionnaire link was shared by email to the potential participants through the software Google Forms (www.forms.google.com). Prior to distributing the e-survey, the questionnaire was developed by reviewing the most recent literature and pre-tested by the authors, evaluating its completion time, design, questions order, attractiveness, syntax, clarity, logic, correct question type, and response format. Furthermore, the questionnaire was reviewed by two independent experts with experience in methodology and in the areas of health and exercise, and pre-tested in a representative sample of ten Rink Hockey practitioners where they were able to comment and suggest improvements. The e-survey took approximately 20 min to complete and included 53 close-ended questions, divided into two main stages (33 sport and sociodemographic-related items, and 20 health disorders and injuries-related items). The sport and sociodemographic-related items were mandatory, whereas injury-specific questions appeared conditionally based on reported injuries (no data imputation was performed). To ensure a proper questionnaire filling, definitions and examples were given throughout the items, helping to contextualize the readers. For instance, regarding injuries, an adaptation of the IOC/STROBE-SIIS consensus was utilized, defining an injury as any tissue damage or impairment of the athlete’s normal physical function that prevented full participation in subsequent Rink Hockey training sessions/competitions or required medical attention [19].

### 2.3. Statistical Analysis

Data from the online questionnaire were entered into a protected database, from which the data were determined and displayed in tabular and graphic formats, using the Microsoft Excel (Microsoft Corp, Redmond, Washington, DC, USA) and IBM SPSS 28.0 (International Business Machines Corporation, Statistical Package for the Social Sciences, Armonk, NY, USA) software. The injury prevalence was determined by dividing the total number of participants who experienced at least one injury by the total number of athletes and multiplied by 100. In addition, the injury rate was calculated by dividing the total number of injuries reported in the last 12 months by the total exposure time to risk (defined as 1000 h). The total exposure time was estimated by adding the average training sessions per week times the average training duration in hours over the session weeks and the total number of games in the season times the average game duration in hours. This calculation was performed at the individualized level for both the entire sample and for the subgroup of injured athletes. Furthermore, the average number of injuries per athlete was calculated in two ways: one, by dividing the total number of injuries by the total number of athletes, and another by dividing the total number of injuries by the number of injured athletes [20]. As the data did not follow a normal distribution, independent two-sample non-parametric Mann–Whitney U (ordinal data) and chi-square tests (nominal data) were conducted. Additionally, Spearman’s rho correlations were performed between the variables. The strength of the relationship was evaluated according to the following criteria [21]: 0 to 0.20—negligible; 0.21 to 0.40—weak; 0.41 to 0.60—moderate; 0.61 to 0.80—strong; and 0.81 to 1.00—very strong. Moreover, logistic regression analyses using the enter method were conducted to examine the associations between the Rink Hockey practitioners’ sport and sociodemographic characteristics and injury outcomes. Candidate variables were initially evaluated individually, and exploratory models were subsequently examined across different injury types and localizations. Given the relatively small number of events observed for several injuries, only models considered sufficiently stable were retained. To minimize the risk of overfitting and unstable parameter estimates, multivariable models, including multiple covariates, were not pursued when the number of outcome events was considered insufficient relative to the number of parameters to be estimated. A significance level of 0.05 was used to define whether a model needed to be reported [22]. Odds ratios (ORs) and their 95% confidence intervals (CIs) were determined for each level of the independent variables. Cis, including 1.0, were considered as not statistically significant [23]. The R^2^ values were interpreted as follows [24]: R^2^ < 2%—very weak; 2% ≤ R^2^ < 13%—weak; 13% ≤ R^2^ < 26%—moderate; and R^2^ ≥ 26%—substantial.

## 3. Results

**Figure 1:**
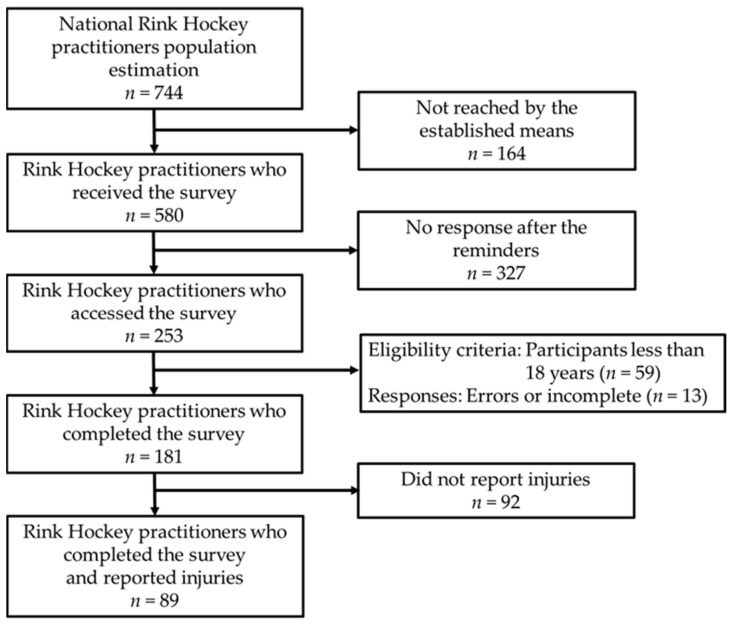
Questionnaire views, participation, and completion.

With the e-survey, 580 Rink Hockey practitioners could be reached and, among them, 253 accessed the questionnaire. Of those, only 181 responses were considered valid. Figure 1 illustrates the study’s flow diagram.

Among the 181 Rink Hockey practitioners who completed the questionnaire, the majority were males (124; 68.5%), with a mean age of 22.3 (±4.3) years, and a BMI of 23.5 (±2.5) kg/m^2^. The participants reported a mean practice experience of 15.8 (±5.0) years. In terms of playing position, the majority occupied the midfielder role (59; 32.6%), followed by forward (51; 28.2%), defender (40; 22.1%), and goalkeeper (31; 17.1%).

Regarding the 2024/2025 competitive level, most competed in the Under-23 category (87; 48.1%), followed by 1st Division Female (56; 30.9%) and 1st Division Male (38; 21.0%). The participants reported engaging in a mean of 3.6 (±1.0) Rink Hockey training sessions per week, totaling 367.2 (±164.0) minutes. Most practitioners trained once daily (159; 87.8%). Additionally, 97 (53.6%) reported practicing additional physical activity, with Gym being the most common (70; 38.7%), at a mean duration of 96.3 (±115.3) minutes per week. During the 2024/2025 season, participants played a mean of 31.9 (±14.9) games, with a mean duration of 30.3 (±11.9) minutes per game.

The majority trained in the Porto region (64; 35.4%), on wooden surfaces (128; 70.7%), in facilities with good surface conditions (126; 69.6%), and without air conditioning (140; 77.3%). Regarding warm-up routines, most participants reported warming up before training and competitions (173; 95.6%), typically for 10–20 min (87; 48.1%), incorporating mainly mobility exercises (162; 25.9%), dynamic stretching (112; 17.9%), and passing exercises (104; 16.6%). Injury prevention was the primary reason for performing warm-up (103; 59.5%). Similarly, 115 (63.5%) reported using cool-down strategies, with the majority spending less than 10 min (86; 47.5%), mainly performing stretching exercises (100; 36.9%). Injury prevention was once again the most reported reason for cool-down among those who practiced it (43; 37.4%).

Most participants were right-handed (130; 71.8%) and used the Azemad Special (45; 24.9%) stick model, with a close blade type (95; 52.5%), a thin shaft (129; 71.3%), and applied grip tape in a continuous pattern (141; 77.9%). Concerning wheels hardness, 92A was the most frequently reported (93; 51.4%). Regarding protective equipment, the majority reported using shin guards (179; 21.8%), followed by knee guards (179; 21.8%), and gloves (179; 21.8%). Concerning staff support, most participants reported having a coach (167; 23.7%), a team manager (134; 19.0%), an assistant coach (98; 13.9%), and a strength and conditioning coach (97; 13.8%), while fewer had access to a physiotherapist (89; 12.6%), massage therapist (49; 7.0%), nurse (39; 5.5%), or medical doctor (20; 2.8%). Detailed data on Rink Hockey practitioners’ sport and sociodemographic characteristics can be found in Table 1.

Of the 181 Rink Hockey practitioners who completed the questionnaire, 89 (49.2%) reported having an injury. Over their years of practicing Rink Hockey, most reported experiencing five or more injuries (27.0%—mean of 2.6 (±2.7) for total sample and 3.3 (±3.1) per injured athlete), with at least one of these occurring in the 2024/2025 season (70.8%—mean of 0.7 (±0.8) the total sample and 1.3 (±0.6) per injured athlete), resulting in an estimated injuries incidence of 3.9 (±5.9) for the total sample and 7.9 (±6.2) per injured athlete, per 1000 h of exposure. Regarding injury localization, the knee was the most affected area (17; 19.1%), followed by the ankle (10; 11.2%), and thigh posterior (8; 9.0%). Concerning injury type, muscle injuries were the most prevalent (23; 25.8%), followed by bone injuries (14; 15.7%), and joint injuries (11; 12.4%).

Most injuries occurred during training (35; 39.3%), followed by competition in the second half (23; 25.8%) and first half (18; 20.2%). Regarding the court zones, the areas surrounding the goal (Zone 5 [18; 20.2%], 6 [22; 24.7%], and 7 [11; 12.4%]) were where most of the injuries occurred. Regarding game set pieces, offensive transition was the most common situation (36; 40.4%), followed by set defense (15; 16.9%), defensive transition (12; 13.5%), and set attack (11; 12.4%). In terms of season timing, most injuries occurred in the mid-season (49; 55.1%), followed by early season (27; 30.3%) and end-season (13; 14.6%). The perceived reasons for injury were predominantly contact with another player (24; 27.0%), overuse (20; 22.5%), and contact with the ball (12; 13.5%).

**Figure 2:**
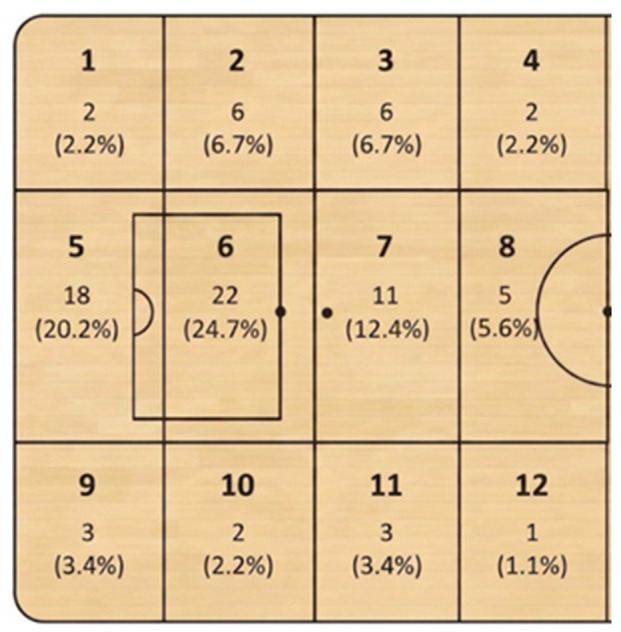
Rink Hockey court division by injuries occurrences and percentages (*n* = 89). Note: A total of eight participants (9.0%) reported being unable to identify a specific court localization where the injury occurred.

**Figure 3:**
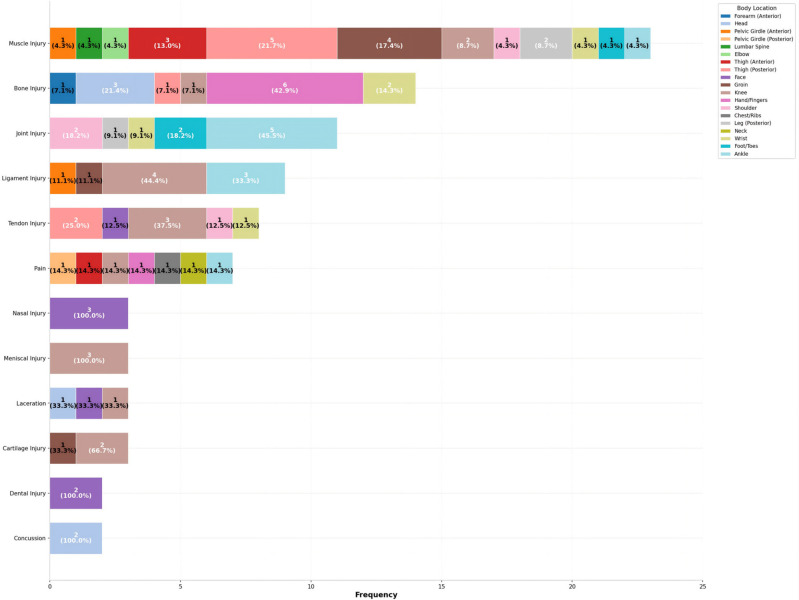
Localization by injury type distribution (*n* = 89).

Regarding return-to-sport duration, injuries were usually of moderate severity, with most practitioners returned within 1–2 weeks (23; 25.8%), 2–3 weeks (22; 24.7%), and 1–3 months (19; 21.3%). The majority of injuries were first-time occurrences (65; 73.0%), while 24 (27.0%) were recurrences. Regarding injury management, physiotherapist intervention was the most common approach (52; 58.4%), followed by rest (38; 42.7%), and active self-management (25; 28.1%). Concerning functional limitations associated with the injury, 60 (67.4%) reported no limitations, while 29 (32.6%) reported limitations with a mean level of 3.8 (±1.7). Similarly, 64 (71.9%) reported no persistent pain, while 25 (28.1%) reported persistent pain with a mean level of 3.8 (±1.5). Fear of injury was reported by 53 (59.6%) practitioners, with a mean level of 5.9 (±2.4), while 36 (40.4%) reported no fear. For more detailed information, see Figure 2 and Figure 3, and Table 2 and Table 3.

When comparing practitioners who reported injuries (89; 49.2%) with those who did not (92; 50.8%), statistically significant differences were found for age (*p* = 0.042; ES = 0.174) and sex (*p* = 0.026; ES = 0.166), with injured participants being older (21.8 (±4.2) vs. 22.9 (±4.3)) and having a higher proportion of females (males: 70 (76.1%) vs. 54 (60.7%); females: 22 (23.9%) vs. 35 (39.5%)). The other variables did not exhibit significant statistical differences (refer to Table 1 and Table S1 for additional details).

It was also found that statistically significant correlations exist between injuries and other variables. Age showed a moderate positive correlation with hockey years (0.826, *p* ≤ 0.001) and a weak positive correlation with total hockey injuries (0.262, *p* ≤ 0.001), as well as with 1-year hockey injuries (0.158, *p* ≤ 0.05), indicating that older players reported more injuries both throughout their careers and in the past year. Hockey years showed a weak positive correlation with total hockey injuries (0.157, *p* ≤ 0.05) and with 1-year hockey injuries (0.157, *p* ≤ 0.05), suggesting that more experienced players reported a higher number of injuries. Additionally, total hockey injuries showed a moderate positive correlation with 1-year hockey injuries (0.548, *p* ≤ 0.001), indicating that athletes with a higher cumulative injury history were also more likely to report recent injuries. For detailed results, see Table 4.

Regarding the logistic regressions, one statistically significant logistic regression model was identified for joint injuries. The model (R^2^ = 0.224; *p* = 0.011) revealed that Rink Hockey practice years were a significant predictor. Compared to practitioners with ≤10 years of experience, those with 11–15 years (OR = 0.116, 95% CI [0.019; 0.695]; *p* = 0.018), 16–19 years (OR = 0.116, 95% CI [0.019; 0.695]; *p* = 0.018), and ≥20 years (OR = 0.056, 95% CI [0.006; 0.534]; *p* = 0.012) of experience, had significantly lower odds of sustaining a joint injury, indicating a dose–response relationship, where longer practice duration was progressively associated with reduced likelihood of joint injury. For more detailed information, see Table 5.

## 4. Discussion

The present study provided a comprehensive epidemiological characterization of senior and under-23 federated Rink Hockey practitioners in Portugal. It was found that approximately half of the surveyed athletes (49.2%) reported at least one injury (mean of 2.6 injuries per career and 0.7 per season), with an estimated incidence of 3.9 ± 5.9 injuries per 1000 h of exposure across the total sample and 7.9 ± 6.2 per 1000 h among injured athletes.

These values are consistent with other studies, yet in the lower bound of the range described in the literature, where values between 3.23 and 14.2 injuries/1000 h have been reported [1]. Furthermore, in the present study, 39.3% of injuries occurred during training and 46.0% during competitive match play. Other studies also found that the majority of injuries occurred in matches (~45–75%, 17.5–36.7 injuries/1000 h in matches vs. ~25–55%, 1.77–7.5 injuries/1000 h in training) [1].

These discrepancies likely reflect methodological differences rather than reporting error [11]. Most studies included in the review used prospective surveillance methods recorded by professionals, whereas the present study employed retrospective self-reports by the athletes. For example, head/face/neck injuries usually represent the third-most important injury area (1.2–20.8%); however, in the present study, these regions combined accounted for only 15.7%. In fact, in a cross-sectional study performed during the 2019 Rink Hockey World Championship with senior male, senior female, and under-19 athletes from three national teams (Argentina, Portugal, and Spain), injuries were reported by the medical staff, and the most frequently reported injuries were head contusions (25%) [25], which may impact the overall study and the suggestions to reduce these injuries (such as helmet requirement for outfield players).

The present study identified age, experience, and sex as the most significant variables associated with injury occurrence. Notably, age and experience can exert both detrimental and protective influences on injury occurrence. On the one hand, it is expected that the older the Rink Hockey practitioner, the more years of practice they accumulate (0.826; *p* ≤ 0.001), and the greater their exposure to the risks inherent to the sport. In support of this, statistically significant positive correlations were found between age and both career (0.262; *p* ≤ 0.001) and one-season (0.158; *p* ≤ 0.05) injuries, as well as between experience and both career (0.157; *p* ≤ 0.05) and one-season (0.157; *p* ≤ 0.05) injuries. In the present study, correlation analysis demonstrated that cumulative career injury burden was a moderately positive factor correlated with one-season injury occurrence (0.548; *p* ≤ 0.001), consistent with the established concept that prior injury history is among the strongest predictors of future injury risk in musculoskeletal epidemiology (often associated with premature return-to-sport, incomplete tissue healing, or residual biomechanical/neuromuscular deficits) [26]. On the other hand, injury recurrence was relatively low, representing only 27.0% of cases in the present study. In fact, a dose–response protective effect of Rink Hockey experience against joint injuries appears to exist, as athletes with 11–15 years (OR = 0.116, 95% CI: 0.019–0.695), 16–19 years (OR = 0.116, 95% CI: 0.019–0.695) and ≥20 years (OR = 0.056, 95% CI: 0.006–0.534) of experience had substantially lower odds of sustaining joint injuries compared to those with ≤10 years of experience.

This finding is particularly important given that lower-extremity joint injuries were among the most frequently reported in the literature among Rink Hockey athletes (joint: 11.3–44.4%; lower limbs: 34.1–65.4%) [1]. In the present study, knee injuries ranked first (19.1%) and ankle injuries second (11.2%) in frequency. From a biomechanical standpoint, the predominance of lower-extremity joint injuries in Rink Hockey is mechanistically plausible. Athletes perform repeated unilateral loading, rapid directional changes, and sudden accelerations and decelerations on quad roller skates, generating high valgus and torsional moments at the lower extremities. The skating push-off in Rink Hockey involves extended periods of single-leg stance with the knee in moderate flexion, placing sustained demands on the medial knee stabilizers and the quadriceps and hamstring co-contraction mechanisms. Additionally, the nature of this contact sport, with frequent high-velocity collisions, represents a primary risk scenario for joint injury, as some of these collisions may surpass the physiological capacity of the structures. In other similar sports that share these characteristics, such as ice hockey, knee medial collateral ligament tears and muscle adductor/abdominal strains have similarly been identified as the most prevalent injuries [27]. The parallel patterns across different skating-based hockey modalities support the notion that the skating action itself, rather than the playing surface, is a primary driver of lower-extremity injury risk in this class of sports. This progressive protective effect may reflect neuromuscular adaptations, refined sport-specific technical skills, and improved movement economy achieved through years of sport-specific technical practice [2,28]. Alternatively, it may partly reflect survivorship bias and the selective retention of healthier athletes, whereby athletes who have sustained recurrent joint injuries retire from competitive participation before accumulating greater years of experience. Nonetheless, the protective dose–response observed herein has potential implications for the design of early-career injury prevention programs and sport-specific preparation, which should prioritize athletes in the first decade of participation.

As already described, a statistically significant difference was found in the sex variable (*p* = 0.026; ES = 0.166), with a higher injury rate observed among female athletes (39.3% injured vs. 23.9% non-injured). In a two-season observational study, Spanish female Rink Hockey players were found to have incidence rates of 2.6/1000 h (95% CI: 2.2–3.1) during training, and 15.2/1000 h (95% CI: 12.2–18.3) during match sessions. The region with the greatest number of injuries was the thigh (18%), followed by the hip/groin (13%) and the knee (12%). Injury incidence was 1.3/1000 h (95% CI: 1.0–1.6) for musculotendinous injuries, 0.85 (95% CI: 0.6–1.0) for ligaments, 0.8 (95% CI: 0.5–1.0) for contusions, 0.3 (95% CI: 0.1–0.4) for bone, and 0.2 (95% CI: 0.08–0.3) for nerve injuries. Seventy-nine percent of injuries had an acute onset, and 21% had a gradual onset [29]. Comparatively, in their male counterparts from the same Spanish championship, an incidence rate of 3.1 (95% CI: 2.66–3.53) was found in training and 23 (95% CI: 15.6–30.44) during games. The most injured location was the hip/groin (15.8%), followed by the shoulder (12.9%), thigh (9.9%), and head/neck (8.9%). Injury incidence was 1.25 (95% CI: 0.79–1.72) for musculotendinous injuries, 0.76 (95% CI: 0.4–1.12) for ligaments, 0.26 (95% CI: 0.05–0.48) for bone, and 0.04 (95% CI: 0–0.13) for nerve injuries. Seventy-two percent of injuries had an acute onset, and 9.90% had an insidious onset [2].

In the two studies previously explored, the most important difference lies in the anatomical regions reported: among female Rink Hockey athletes, the thigh (18%) was most affected, followed by the hip/groin (13%) and the knee (12%), whereas among males, the hip/groin (15.8%) predominated, followed by the shoulder (12.9%) and thigh (9.9%). These differences may be attributable to hormonal, anatomical, and neuromuscular sex-related factors [30,31]. Given the nature of the sport, players usually perform more than 300 sudden accelerations and decelerations each session, posing high demands on the main muscle groups of the lower limb, particularly the rectus femoris [32]. Additionally, the early swing phase of the sprint cycle, during the transition from maximal hip extension to maximum hip and knee flexion, combined with high eccentric activity, can impose excessive tension on the anterior thigh muscle group, leading to possible injuries [33]. As female athletes generally exhibit lower force-production capacity [30,31], this may partially explain the sex-specific differences observed herein. This may also explain the predominant injury type encountered. Muscle injuries were the most frequently reported injury type in the present study (25.8%), both in male and female athletes (27.8% and 22.9%, respectively). This finding is consistent across the existing Rink Hockey literature, where a systematic review reported overall muscle-tendinous injuries of 25 to 43.7% [1].

Furthermore, in the present study, bone injuries were the second-most frequent type (15.7%), followed by joint injuries (12.4%). This may reflect the nature of this contact sport and may be particularly relevant for female athletes. In the total sample of the present study, contact with another player was the most frequently reported perceived injury mechanism (27.0%), followed by overuse (22.5%) and ball contact (13.5%). This distribution does not fully align with the broader Rink Hockey literature, where in one systematic review, overuse was identified as the most common cause, representing approximately 45 to 55%, while direct trauma represented approximately 20 to 40% [1]. However, in the sex subgroup analysis of the present study, it was found that males reported 29.6% of overuse injuries and 24.1% contact with another player, whereas their female counterparts reported 31.4% contact with another player and only 11.4% of overuse injuries. Therefore, as female players generally present less neuromuscular control, strength capacity, and joint laxity [30,31], the nature of the sport—combining high-speed movements and transitions with high contact (particularly during offensive transition (40.4%) in areas surrounding the goal (57.3%)), where moments of high kinetic energy and reduced player control are encountered throughout the game—may require changes recommending proper physical preparation to sustain impacts, rule modifications to severely penalize athletes who commit contact fouls, and implementation of more protective equipment to reduce these injuries in this population [1,26]. As for male athletes, as the most reported mechanism was overuse, this may be associated with the predominance of mid-season injuries reported (55.1%). Increases in injury incidence during mid-season are well documented in team sports and have been attributed to the progressive accumulation of training load and insufficient recovery, with the acute-to-chronic workload ratio emerging as a key modifying factor [34]. As a result, special attention needs to be given to physical conditioning, fatigue monitoring (and interventions to reduce it), and season planning (training and competition volume) in order to reduce these injuries in this population.

Finally, a finding of notable clinical relevance in the present study was the high prevalence of fear of re-injury among injured athletes. Although the majority of injuries in the present study were of moderate severity (with 50.5% of injured athletes recovering within one to three weeks) and were managed conservatively (physiotherapy: 58.4%), the fear of re-injury reported among injured athletes was 59.6%, with a mean score of 5.9 ± 2.4 on a 0–10 scale, alongside persistent pain (28.1%; mean 3.8 ± 1.5) and functional limitations (32.6%; mean 3.8 ± 1.7) following recovery. Fear of re-injury is recognized as the leading psychological barrier to successful recovery after musculoskeletal injury, and has been associated with reduced activity levels, impaired rehabilitation outcomes, and greater re-injury rates [35]. The prevalence observed in the present study substantially exceeds what might be expected based on the relatively moderate severity of most injuries reported, suggesting that even injuries with short recovery durations can exert lasting psychological effects.

### Limitations

The present study has several methodological limitations that should be considered when interpreting the findings: (1) Although the instrument was developed based on previous epidemiological studies, expert review, and pilot testing, formal psychometric validation was not performed (which may have introduced measurement error or affected the accuracy and consistency of some self-reported variables). (2) The retrospective, cross-sectional, self-reported design is susceptible to recall bias, particularly for injuries sustained early in the 12-month recall period (more recent events are systematically more likely to be recalled, which may result in underestimation of early season injuries and of certain injuries—minor or overuse). (3) The use of a non-randomized convenience sample recruited through club communication channels introduces potential selection bias (athletes who sustained injuries may have been more motivated to participate, potentially inflating injury prevalence estimates, whereas athletes who withdrew from competition due to injury may not have been reachable through active club channels, introducing competing biases in the opposite direction). (4) Exposure time was estimated retrospective from self-reported training frequency and game participation, rather than measured prospectively (this may introduce imprecision in incidence rate calculations, particularly given the substantial variability in training duration (SD: ±164.0 min/week) reported across participants). (5) Due to the retrospective nature of the study, injury incidence was estimated from individual self-reported exposure data rather than aggregate surveillance rates (which may limit direct comparability with prospective epidemiological studies and introduce additional measurement variability). (6) The sample size of 181 valid responses, while representing a reasonable proportion of the eligible federated population of 744 athletes, falls below the pre-specified target of 254 responses (which may have limited the statistical power to detect small-to-moderate associations, increasing the risk of Type II error and reliability of subgroup comparisons and exploratory regression analyses, particularly for rarer injury categories, specific playing positions, and sex-stratified analyses). (7) The study combined under-23 and senior athletes, and while this reflects the real structure of Portuguese Rink Hockey, it may have limited age-category-specific patterns. (8) Injury classification relied on participant self-report without medical adjudication, which may have led to misclassification of specific diagnoses. (9) The study was conducted during a single competitive season, and findings may not be fully generalizable to other seasons, which may differ in terms of competitive calendars, weather conditions, or team composition. (10) The sample consisted exclusively of Portuguese federated senior and under-23 Rink Hockey players, a population exposed to sport-specific demands related to skating, stick handling, player contact, and competition structure. Consequently, the observed injury patterns and associated factors may not be directly transferable to athletes from other countries, competitive systems, age groups, or sporting disciplines. Future multicenter and international studies are needed to determine the extent to which these findings apply to other elite athletic populations.

## 5. Conclusions

Portuguese Rink Hockey practitioners reported a substantial lifetime burden of sport-related injuries: 49.2% of respondents have experienced at least one injury (mean 2.6 ± 2.7 injuries per athlete in the total sample; 3.3 ± 3.1 per injured athlete), with an estimated incidence of 3.9 ± 5.9 injuries/1000 h for the total sample (7.9 ± 6.2/1000 h among injured athletes). Muscle (25.8%), bone (15.7%), and joint (12.4%) injuries were the most common types, while the knee (19.1%), ankle (11.2%), and posterior thigh (9.0%) were the most frequently affected anatomical areas. Injuries occurred predominantly during matches (46.0%), with offensive transition (40.4%) being the most hazardous game situation, and the areas surrounding the goal accounting for the majority of injury locations (57.3%). Contact with another player (27.0%) and overuse (22.5%) were the most perceived causes. Statistical analyses identified age (older athletes) and female sex as significant injury reporting factors (*p* ≤ 0.05), while greater Rink Hockey experience (≥11 years) was protective against joint injuries (OR ranging from 0.056 to 0.116). The majority of injuries were first-time occurrences (73.0%), of moderate severity (recovering within 1–3 weeks in 50.5% of cases), and managed primarily through physiotherapy (58.4%). Notably, 59.6% of injured athletes reported a fear of re-injury (mean level 5.9/10), and 28.1% and 32.6% experienced persistent pain and functional limitation after recovery, respectively.

Overall, the findings of the present study provide important implications for clinical practice, athlete education, injury prevention, and future research in Rink Hockey. The predominance of knee injuries, the substantial proportion of female athletes among injured players, and the high prevalence of musculotendinous and overuse injuries support the implementation of structured injury prevention exercise programs focused on neuromuscular coordination, dynamic stability, movement control, balance/proprioceptive training, strength-training (especially eccentric hamstring exercises), and correction of lower-limb strength asymmetries. In parallel, the high frequency of contact-related injuries occurring during offensive transition phases and within goal-area zones highlights the need for stricter rule enforcement regarding illegal obstruction, stick use, and charging, alongside the optimization of protective equipment standards. This is particularly relevant given the absence of mandatory helmet use for outfield players and the non-negligible proportion of head and facial injuries observed, most commonly resulting from ball contact. The significant prevalence of overuse injuries further emphasizes the importance of systematic load monitoring, workload regulation, and structured recovery strategies, particularly during the mid-season period, identified as the peak injury phase. Moreover, the high prevalence of fear of re-injury following recovery underscores the necessity of integrating psychological support and validated psychometric monitoring into rehabilitation pathways. Decisions should therefore incorporate both physical and psychological readiness assessments rather than relying exclusively on symptom resolution or time-based criteria. The limited access to multidisciplinary healthcare professionals observed in this cohort represents an important structural limitation, with potential consequences for both acute injury management and long-term rehabilitation quality. Expanding access to physiotherapists, nurses, and medical doctors should therefore be prioritized within Portuguese Rink Hockey governance structures.

Future epidemiological studies should prioritize standardized prospective injury-surveillance designs with individual-level exposure tracking, and should further investigate sex-specific and position-specific injury patterns, game situations, protective equipment strategies, and the injury-related psychological dimensions of Rink Hockey. Finally, intervention studies are needed to determine whether neuromuscular training, workload management, and rules changing can effectively reduce the incidence of injuries in this sport.

## Figures and Tables

**Table 1:** Rink Hockey practitioners’ sport and sociodemographic characteristics.

Variable	Total (*n* = 181 (100%)) *n* (%)	Unreported Injury (*n* = 92 (50.8%)) *n* (%)	Reported Injury (*n* = 89 (49.2%)) *n* (%)	*p* Injury vs. No Injury
Age (years)				0.042 *
*≤20*	74 (40.9)	43 (46.7)	31 (34.8)	
*21–25*	71 (39.2)	37 (40.2)	34 (38.2)	
*≥26*	36 (19.9)	12 (13.0)	24 (27.0)	
*Mean (SD)*	22.3 (4.3)	21.8 (4.2)	22.9 (4.3)	
Sex				0.026 **
*Female*	57 (31.5)	22 (23.9)	35 (39.3)	
*Male*	124 (68.5)	70 (76.1)	54 (60.7)	
BMI (kg/m^2^)				0.361 *
*≤20*	12 (6.6)	7 (7.6)	5 (5.6)	
*21–24*	113 (62.4)	61 (66.3)	52 (58.4)	
*≥25*	56 (30.9)	24 (26.1)	32 (36.0)	
*Mean (SD)*	23.5 (2.5)	23.3 (2.4)	23.6 (2.7)	
Practice Years				0.052 *
*≤10*	28 (15.5)	14 (15.2)	14 (15.7)	
*11–15*	64 (35.4)	39 (42.4)	25 (28.1)	
*16–19*	53 (29.3)	28 (30.4)	25 (28.1)	
*≥20*	36 (19.9)	11 (12.0)	25 (28.1)	
*Mean (SD)*	15.8 (5.0)	15.0 (4.6)	16.5 (5.2)	
2024/2025 Competitive Level				0.561 **
*1st Division Male*	38 (21.0)	18 (19.6)	20 (22.5)	
*1st Division Female*	56 (30.9)	22 (23.9)	34 (38.2)	
*Under-23*	87 (48.1)	66 (71.7)	21 (23.6)	
Playing Position				0.470 **
*Forward*	51 (28.2)	24 (26.1)	27 (30.3)	
*Midfielder*	59 (32.6)	27 (29.3)	32 (36.0)	
*Defender*	40 (22.1)	22 (23.9)	18 (20.2)	
*Goalkeeper*	31 (17.1)	19 (20.7)	12 (13.5)	
Rink Hockey Weekly Training				0.376 *
*≤2*	13 (7.2)	7 (7.6)	6 (6.7)	
*3–4*	138 (76.2)	69 (75)	69 (77.5)	
*≥5*	30 (16.6)	16 (17.4)	14 (15.7)	
*Mean (SD)*	3.6 (1.0)	3.6 (1.0)	3.5 (1.0)	
Rink Hockey Weekly Training (minutes)				0.985 *
*≤200*	28 (15.5)	13 (14.1)	15 (16.9)	
*201–399*	88 (48.6)	48 (52.2)	40 (44.9)	
*≥400*	65 (35.9)	31 (33.7)	34 (38.2)	
*Mean (SD)*	367.2 (164.0)	363.4 (150.0)	371.2 (178.1)	
Rink Hockey Daily Training				0.412 *
*1*	159 (87.8)	79 (85.9)	80 (89.9)	
*2*	8 (4.4)	5 (5.4)	3 (3.4)	
*3*	13 (7.2)	7 (7.6)	6 (6.7)	
*4*	1 (0.6)	1 (1.1)	0 (0.0)	
*Mean (SD)*	1.2 (0.6)	1.2 (0.6)	1.2 (0.5)	
Additional Physical Activity				0.227 **
*Gym*	70 (38.7)	31 (33.7)	39 (43.8)	
*Padel*	7 (3.9)	5 (5.4)	2 (2.2)	
*Running*	6 (3.3)	5 (5.4)	1 (1.1)	
*Swimming*	2 (1.1)	0 (0.0)	2 (2.2)	
*Cycling*	2 (1.1)	1 (1.1)	1 (1.1)	
*Other*	8 (4.4)	5 (5.4)	3 (3.4)	
*More than one*	2 (1.1)	1 (1.1)	1 (1.1)	
*Do not practice*	84 (46.4)	43 (46.7)	41 (46.1)	
Additional Physical Activity (minutes)				0.912 *
*≤100*	19 (19.6%)	10 (20.4%)	9 (18.8%)	
*101–199*	50 (51.5%)	24 (49.0%)	26 (54.2%)	
*≥200*	28 (28.9%)	15 (30.6%)	13 (27.1%)	
*Mean (SD)*	96.3 (115.3)	98.5 (117.6)	94.1 (113.4)	
2024/2025 Season Games				0.674 *
*≤20*	43 (23.8)	27 (29.3)	16 (18.0)	
*21–39*	93 (51.4)	40 (43.5)	53 (59.6)	
*≥40*	45 (24.9)	25 (27.2)	20 (22.5)	
*Mean (SD)*	31.9 (14.9)	31.7 (16.3)	32.2 (13.4)	
2024/2025 Season Games (minutes)				0.533 *
*≤20*	47 (26.0)	30 (32.6)	17 (19.1)	
*21–39*	80 (44.2)	34 (37.0)	46 (51.7)	
*≥40*	54 (29.8)	28 (30.4)	26 (29.2)	
*Mean (SD)*	30.3 (11.9)	29.4 (13.4)	31.1 (10.2)	
Dominant Hand				0.722 **
*Right*	130 (71.8)	65 (70.7)	65 (73.0)	
*Left*	51 (28.2)	27 (29.3)	24 (27.0)	
Stick Model				0.143 **
*Azemad Special*	45 (24.9)	27 (29.3)	18 (20.2)	
*Azemad RV66*	22 (12.2)	10 (10.9)	12 (13.5)	
*Azemad Plus*	19 (10.5)	10 (10.9)	9 (10.1)	
*Azemad GT10*	18 (9.9)	13 (14.1)	5 (5.6)	
*Azemad 100 Special*	11 (6.1)	5 (5.4)	6 (6.7)	
*Azemad Strong*	11 (6.1)	7 (7.6)	4 (4.5)	
*Azemad Flash*	7 (3.9)	2 (2.2)	5 (5.6)	
*Azemad Azex Special*	4 (2.2)	0 (0.0)	4 (4.5)	
*Jet Special Force*	4 (2.2)	3 (3.3)	1 (1.1)	
*Azemad Compact Plus*	3 (1.7)	2 (2.2)	1 (1.1)	
*Azemad Jumbo*	3 (1.7)	1 (1.1)	2 (2.2)	
*Azemad RV66 Elite*	3 (1.7)	0 (0.0)	3 (3.4)	
*Kumbre*	3 (1.7)	0 (0.0)	3 (3.4)	
*Azemad Azex Elite*	2 (1.1)	2 (2.2)	0 (0.0)	
*Azemad Keeper Carbon*	2 (1.1)	1 (1.1)	1 (1.1)	
*Azemad Red Bull HN*	2 (1.1)	1 (1.1)	1 (1.1)	
*Other*	20 (11.0)	7 (7.6)	13 (14.6)	
*Do not know*	2 (1.1)	1 (1.1)	1 (1.1)	
Stick Blade Type				0.081 **
*Close*	95 (52.5)	55 (59.8)	40 (44.9)	
*Open*	38 (21.0)	14 (15.2)	24 (27.0)	
*Do not know*	48 (26.5)	23 (25.0)	25 (28.1)	
Shaft Type				0.483 **
*Thin*	129 (71.3)	63 (68.5)	66 (74.2)	
*Thick*	40 (22.1)	21 (22.8)	19 (21.3)	
*Do not know*	12 (6.6)	8 (8.7)	4 (4.5)	
Grip Tape Application Pattern				0.201 **
*Continuous*	141 (77.9)	77 (83.7)	64 (71.9)	
*Interrupted*	17 (9.4)	5 (5.4)	12 (13.5)	
*Figure-eight*	8 (4.4)	4 (4.3)	4 (4.5)	
*Do not apply*	15 (8.3)	6 (6.5)	9 (10.1)	
Protective Equipment Used				0.552 **
*Shin guards*	179 (21.8%)	91 (21.0%)	88 (22.6%)	
*Knee guards*	179 (21.8%)	91 (21.0%)	88 (22.6%)	
*Gloves*	179 (21.8%)	91 (21.0%)	88 (22.6%)	
*Pelvic protector*	118 (14.4%)	66 (15.2%)	52 (13.4%)	
*Chest guard*	46 (5.6%)	25 (5.8%)	21 (5.4%)	
*Elbow guards*	39 (4.7%)	23 (5.3%)	16 (4.1%)	
*Helmet*	32 (3.9%)	19 (4.4%)	13 (3.3%)	
*Throat guards*	30 (3.6%)	18 (4.2%)	12 (3.1%)	
*Mouthguard*	17 (2.1%)	8 (1.8%)	9 (2.3%)	
*Padded shorts*	3 (0.4%)	1 (0.2%)	2 (0.5%)	
Wheels Hardness				0.440 **
*88A*	13 (7.2)	5 (5.4)	8 (9.0)	
*90A*	17 (9.4)	7 (7.6)	10 (11.2)	
*92A*	93 (51.4)	45 (48.9)	48 (53.9)	
*94A*	10 (5.5)	6 (6.5)	4 (4.5)	
*Do not know*	48 (26.5)	29 (31.5)	19 (21.3)	
Training Facility Location				0.850 **
*Porto*	64 (35.4)	35 (38.0)	29 (32.6)	
*Lisboa*	43 (23.8)	20 (21.7)	23 (25.8)	
*Aveiro*	37 (20.4)	19 (20.7)	18 (20.2)	
*Coimbra*	18 (9.9)	9 (9.8)	9 (10.1)	
*Braga*	5 (2.8)	3 (3.3)	2 (2.2)	
*Santarém*	5 (2.8)	1 (1.1)	4 (4.5)	
*Setúbal*	4 (2.2)	3 (3.3)	1 (1.1)	
*Leiria*	3 (1.7)	1 (1.1)	2 (2.2)	
*Viana do Castelo*	2 (1.1)	1 (1.1)	1 (1.1)	
Training Facility Surface Condition				0.411 **
*Good*	126 (69.6)	67 (72.8)	59 (66.3)	
*Powdery*	31 (17.1)	11 (12.0)	20 (22.5)	
*Irregular*	18 (9.9)	10 (10.9)	8 (9.0)	
*Slippery*	3 (1.7)	2 (2.2)	1 (1.1)	
*Do not know*	3 (1.7)	2 (2.2)	1 (1.1)	
Training Facility Surface Type				0.224 **
*Wood*	128 (70.7)	69 (75.0)	59 (66.3)	
*Synthetic*	19 (10.5)	6 (6.5)	13 (14.6)	
*Concrete*	17 (9.4)	7 (7.6)	10 (11.2)	
*Do not know*	17 (9.4)	10 (10.9)	7 (7.9)	
Training Facility Air conditioning				0.461 **
*Yes*	33 (18.2)	20 (21.7)	13 (14.6)	
*No*	140 (77.3)	68 (73.9)	72 (80.9)	
*Do not know*	8 (4.4)	4 (4.3)	4 (4.5)	
Warm-up Before Training/Competitions				0.504 *
*Yes, <10 min*	50 (27.6)	25 (27.2)	25 (28.1)	
*Yes, 10–20 min*	87 (48.1)	41 (44.6)	46 (51.7)	
*Yes, 21–30 min*	24 (13.3)	12 (13.0)	12 (13.5)	
*Yes, >30 min*	12 (6.6)	9 (9.8)	3 (3.4)	
*No warm-up*	8 (4.4)	5 (5.4)	3 (3.4)	
Warm-up Exercises Used				0.517 **
*Mobility exercises*	162 (25.9%)	80 (25.2%)	82 (26.7%)	
*Dynamic stretching*	112 (17.9%)	61 (19.2%)	51 (16.6%)	
*Passing exercises*	104 (16.6%)	51 (16.0%)	53 (17.3%)	
*Static stretching*	62 (9.9%)	34 (10.7%)	28 (9.1%)	
*Core exercises*	52 (8.3%)	29 (9.1%)	23 (7.5%)	
*Sprints*	44 (7.0%)	22 (6.9%)	22 (7.2%)	
*Rink skating*	43 (6.9%)	21 (6.6%)	22 (7.2%)	
*Strengthening exercises*	29 (4.6%)	12 (3.8%)	17 (5.5%)	
*Proprioceptive exercises*	17 (2.7%)	8 (2.5%)	9 (2.9%)	
Reasons to Perform Warm-up				0.810 **
*Injury prevention*	103 (59.5)	49 (56.3)	54 (62.8)	
*Routine*	34 (19.7)	18 (20.7)	16 (18.6)	
*Fitness/Performance*	22 (12.7)	12 (13.8)	10 (11.6)	
*Concentration*	12 (6.9)	6 (6.9)	6 (7.0)	
*Nervousness reduction*	1 (0.6)	1 (1.1)	0 (0.0)	
*Other*	1 (0.6)	1 (1.1)	0 (0.0)	
Cool-down After Training/Competitions				0.823 *
*Yes, <10 min*	86 (47.5)	46 (50.0)	40 (44.9)	
*Yes, 10–20 min*	23 (12.7)	8 (8.7)	15 (16.9)	
*Yes, 21–30 min*	2 (1.1)	1 (1.1)	1 (1.1)	
*Yes, >30 min*	4 (2.2)	3 (3.3)	1 (1.1)	
*No cool-down*	66 (36.5)	34 (37.0)	32 (36.0)	
Cool-down Strategies Used				0.334 **
*Stretching*	100 (36.9%)	54 (40.6%)	46 (33.3%)	
*Nutrition/Supplementation*	35 (12.9%)	16 (12.0%)	19 (13.8%)	
*Massage*	31 (11.4%)	13 (9.8%)	18 (13.0%)	
*Criotherapy*	29 (10.7%)	10 (7.5%)	19 (13.8%)	
*Active recovery*	21 (7.7%)	6 (4.5%)	15 (10.9%)	
*Passive recovery/Rest*	11 (4.1%)	6 (4.5%)	5 (3.6%)	
*Massage Gun*	10 (3.7%)	5 (3.8%)	5 (3.6%)	
*Foam roller*	10 (3.7%)	6 (4.5%)	4 (2.9%)	
*Compression*	8 (3.0%)	3 (2.3%)	5 (3.6%)	
*Hydrotherapy*	6 (2.2%)	5 (3.8%)	1 (0.7%)	
*Sauna*	6 (2.2%)	5 (3.8%)	1 (0.7%)	
*Electric stimulation*	3 (1.1%)	3 (2.3%)	0 (0.0%)	
*Hyperbaric therapy*	1 (0.4%)	1 (0.8%)	0 (0.0%)	
Reasons to Perform Cool-down				0.346 **
*Injury prevention*	43 (37.4)	25 (43.1)	18 (31.6)	
*Fatigue reduction*	32 (27.8)	11 (19.0)	21 (36.8)	
*Well-being and comfort*	20 (17.4)	10 (17.2)	10 (17.5)	
*Relaxation*	12 (10.4)	8 (13.8)	4 (7.0)	
*Routine*	8 (7.0)	4 (6.9)	4 (7.0)	
Staff				0.573 **
*Coach*	167 (23.7%)	85 (23.5%)	82 (23.9%)	
*Team manager*	134 (19.0%)	70 (19.4%)	64 (18.7%)	
*Assistant coach*	98 (13.9%)	46 (12.7%)	52 (15.2%)	
*Strength and conditioning coach*	97 (13.8%)	47 (13.0%)	50 (14.6%)	
*Physiotherapist*	89 (12.6%)	48 (13.3%)	41 (12.0%)	
*Massage therapist*	49 (7.0%)	24 (6.6%)	25 (7.3%)	
*Nurse*	39 (5.5%)	23 (6.4%)	16 (4.7%)	
*Medical doctor*	20 (2.8%)	10 (2.8%)	10 (2.9%)	
*Sports physiologist*	7 (1.0%)	5 (1.4%)	2 (0.6%)	
*None*	4 (0.6%)	3 (0.8%)	1 (0.3%)	

Note: bold—significant statistical difference. Competitive level, protective equipment, warm-up strategies, cool-down strategies, and staff variables were of multiple selections, so percentages presented were calculated over the total responses; * Mann–Whitney U; ** chi-square test.

**Table 2:** Rink Hockey practitioner’s reported injuries characterization (*n* = 89).

Variable	*n* (%)
Total nº of injuries	
*1*	10 (11.2)
*2*	21 (23.6)
*3*	18 (20.2)
*4*	16 (18.0)
*≥5*	24 (27.0)
*Mean total sample (SD)*	2.6 (2.7)
*Mean per injured athlete (SD)*	3.3 (3.1)
Season 2024/2025 nº of injuries	
*1*	63 (70.8)
*2*	22 (24.7)
*3*	4 (4.5)
*Mean total sample (SD)*	0.7 (0.8)
*Mean per injured athlete (SD)*	1.3 (0.6)
Incidence (1000 h)	
*Mean total sample (SD)*	3.9 (5.9)
*Mean per injured athlete (SD)*	7.9 (6.2)
Injury localization	
*Head*	6 (6.7)
*Face*	7 (7.9)
*Neck*	1 (1.1)
*Chest/Ribs*	1 (1.1)
*Lumbar Spine*	1 (1.1)
*Shoulder*	4 (4.5)
*Elbow*	1 (1.1)
*Forearm (anterior)*	1 (1.1)
*Wrist*	5 (5.6)
*Hand/Fingers*	7 (7.9)
*Pelvic Girdle (anterior)*	2 (2.2)
*Pelvic Girdle (posterior)*	1 (1.1)
*Groin*	6 (6.7)
*Thigh (anterior)*	5 (5.6)
*Thigh (posterior)*	8 (9.0)
*Knee*	17 (19.1)
*Lower Leg (posterior)*	3 (3.4)
*Ankle*	10 (11.2)
*Foot/Toes*	3 (3.4)
Injury type	
*Muscle Injury*	23 (25.8)
*Bone Injury*	14 (15.7)
*Joint Injury*	11 (12.4)
*Ligament Injury*	9 (10.1)
*Tendon Injury*	9 (10.1)
*Pain*	7 (7.9)
*Laceration/Abrasion/Bleeding*	3 (3.4)
*Cartilage Injury*	3 (3.4)
*Meniscal Injury*	3 (3.4)
*Nasal Injury*	3 (3.4)
*Concussion*	2 (2.2)
*Dental Injury*	2 (2.2)
Injury occurrence situation	
*Warm-up (training)*	1 (1.1)
*During training*	35 (39.3)
*Cool-down (training)*	1 (1.1)
*Warm-up (competition)*	4 (4.5)
*During competition (1st half)*	18 (20.2)
*During competition (2nd half)*	23 (25.8)
*Cool-down (competition)*	2 (2.2)
*Other*	5 (5.6)
Injury game set pieces situations	
*Offensive transition*	36 (40.4)
*Set defense*	15 (16.9)
*Defensive transition*	12 (13.5)
*Set attack*	11 (12.4)
*Warm-up*	6 (6.7)
*Penalty/Direct Free Hit*	2 (2.2)
*Unknown*	7 (7.9)
Injury in-season	
*Early season*	27 (30.3)
*Mid-season*	49 (55.1)
*End-season*	13 (14.6)
Perceived injury reason	
*Contact with another player*	24 (27.0)
*Overuse*	20 (22.5)
*Contact with ball*	12 (13.5)
*Fatigue*	5 (5.6)
*Incorrect technical gesture—stopping*	5 (5.6)
*Contact with infrastructure*	3 (3.4)
*Fall*	3 (3.4)
*Protective equipment misplacement*	2 (2.2)
*Incorrect technical gesture—spinning*	2 (2.2)
*Contact with stick*	2 (2.2)
*Inadequate warm-up*	1 (1.1)
*Incorrect technical gesture—shooting*	1 (1.1)
*Incorrect technical gesture—passing*	1 (1.1)
*Unknown*	8 (9.0)
Return-to-sport duration	
*<1 week*	11 (12.4)
*1–2 weeks*	23 (25.8)
*2–3 weeks*	22 (24.7)
*1–3 months*	19 (21.3)
*4–6 months*	12 (13.5)
*7–12 months*	2 (2.2)
Injury history	
*First time*	65 (73.0)
*Recurrence*	24 (27.0)
Presence of functional limitations associated with injury	
*No*	60 (67.4)
*Yes (level [0–10]):*	29 (32.6)
*1*	2 (6.9)
*2*	7 (24.1)
*3*	4 (13.8)
*4*	5 (17.2)
*5*	4 (13.8)
*6*	6 (20.7)
*7*	1 (3.4)
*Mean (SD)*	3.8 (1.7)
Persistence pain associated with injury	
*No*	64 (71.9)
*Yes (level [0–10]):*	25 (28.1)
*2*	6 (24.0)
*3*	6 (24.0)
*4*	4 (16.0)
*5*	5 (20.0)
*6*	2 (12.0)
*7*	1 (4.0)
*Mean (SD)*	3.8 (1.5)
Fear of injury	
*No*	36 (40.4)
*Yes (level [0–10]):*	53 (59.6)
*1*	1 (1.9)
*2*	4 (7.5)
*3*	5 (9.4)
*4*	3 (5.7)
*5*	13 (24.5)
*6*	5 (9.4)
*7*	7 (13.2)
*8*	8 (15.1)
*9*	2 (3.8)
*10*	5 (9.4)
*Mean (SD)*	5.9 (2.4)
Injury management	
*Physiotherapist*	52 (58.4)
*Rest*	38 (42.7)
*Active self-management*	25 (28.1)
*Physician (medication)*	18 (20.2)
*Non-conventional medicine*	15 (16.9)
*Self-Medication/Supplementation*	8 (9.0)
*Physician (surgery)*	8 (9.0)
*Physician (injection)*	3 (3.4)
*Speech therapy*	1 (1.1)
*No intervention*	5 (5.6)

**Table 3:** Rink Hockey practitioners’ variables by injury type and localization (*n* = 89).

**Injury** **Localization**	**Perceived Reason**																							
	*Equipment Misplacement*		*Ball Contact*	*Infrastructure Contact*		*Player Contact*	*Inadequate Activation*		*Shot*	*Spin*			*Stop*	*Pass*		*Stick Hit*		*Fall*		*Overuse*		*Fatigue*		*Unknown*
*Head*	-		5	-		1	-		-	-			-	-		-		-		-		-		-
*Face*	-		5	-		2	-		-	-			-	-		-		-		-		-		-
*Neck*	-		1	-			-		-	-			-	-		-		-		-		-		-
*Chest/Ribs*	-		-	-		1	-		-	-			-	-		-		-		-		-		-
*Lumbar Spine*	-		-	-			-		-	-			-	-		-		-		-		1		-
*Shoulder*	-		-	-		1	-		-	-			-	-		-		1		-		-		1
*Elbow*	-		-	-			-		-	-			-	-		-		-		-		-		1
*Forearm (anterior)*	-		-	-		1	-		-	-			-	-		-		-		-		-		-
*Wrist*	-		-	1			-		-	-			1	-		-		1		2		-		-
*Hand/Fingers*	-		1	-		3	-		-	-			-	-		2		-		-		-		-
*Pelvic Girdle (anterior)*	-		-	-			-		-	1			-	-		-		-		1		-		-
*Pelvic Girdle (posterior)*	-		-	-			-		-	-			-	-		-		-		1		-		-
*Groin*	-		-	-		2	-		-	-			1	-		-		-		2		-		1
*Thigh (anterior)*	-		-	-		1	-		-	-			-	-		-		-		3		1		-
*Thigh (posterior)*	1		-	-		1	-		-	-			1	-		-		1		4		-		-
*Knee*	1		-	-		5	-		1	-			-	1		-		-		5		-		4
*Lower Leg (posterior)*	-		-	-			-		-	-			-	-		-		-		1		2		-
*Ankle*	-		-	-		5	-		-	1			2	-		-		-		-		1		1
*Foot/Toes*	-		-	1		1	-		-	-			-	-		-		-		1		-		-
Injury Type																								
*Bone Injury*	1		3	2		4	-		1	-			1	-		2		-		-		-		-
*Cartilage Injury*	-		-	-		-	-		-	-			-	-		-		-		2		-		1
*Concussion*	-		1	-		1	-		-	-			-	-		-		-		-		-		-
*Dental Injury*	-		1	-		1	-		-	-			-	-		-		-		-		-		-
*Joint Injury*	-		-	1		3	1		-	1			2	-		-		-		1		2		-
*Laceration*	-		1	-		1	-		-	-			-	-		-		-		-		-		1
*Ligament Injury*	1		-	-		4	-		-	-			-	-		-		-		-		-		1
*Meniscal Injury*	-		-	-		-	-		-	-			-	-		-		-		1		-		2
*Muscle Injury*	-		-	-		6	-		-	-			-	2		-		-		9		3		3
*Nasal Injury*	-		3	-		-	-		-	1			-	1		-		-		1		-		-
*Pain*	-		2	-		2	-		-	-			-	-		-		-		3		-		-
*Tendon Injury*	-		1	-		2	-		-	-			-	-		-		3		3		-		-
**Injury** **Localization**	**Situation**								**Season**			**Court Zone**												
	*Warm-Up (Training)*	*Warm-Up (Competition)*	*During Training*	*During Competition (1st Half)*	*During Competition (2nd Half)*	*Cool-Down (Training)*	*Cool-Down (Competition)*	*Other*	*Early*	*Mid*	*End*	*1*	*2*	*3*	*4*	*5*	*6*	*7*	*8*	*9*	*10*	*11*	*12*	*Unknown*
*Head*	-	1	3	1	1	-	-	-	2	3	1	1	-	-	-	-	4	-	1	-	-	-	-	-
*Face*	-	-	1	2	4	-	-	-	1	4	2	-	-	-	-	1	5	1		-	-	-	-	-
*Neck*	-	-	-	1	-	-	-	-	1	-	-	-	-	-	-	-	-	-	1	-	-	-	-	-
*Chest/Ribs*	-	-	1	-	-	-	-	-	-	1	-	-	-	-	-	-	-	1	-	-	-	-	-	-
*Lumbar Spine*	-	1	-	-	-	-	-	-	-	-	1	-	-	-	-	-	-	1	-	-	-	-	-	-
*Shoulder*	-	-	1	1	2	-	-	-	3	1	-	1	-	-	-	1	1	1	-	-	-	-	-	-
*Elbow*	-	-	-	-	-	-	-	1	-	1	-	-	-	-	-	1	-	-	-	-	-	-	-	-
*Forearm (anterior)*	-	-	-	1	-	-	-	-	-	1	-	-	-	-	-	-	-	-	1	-	-	-	-	-
*Wrist*	-	-	2	3	-	-	-	-	2	3	-	-	-	-	-	-	1	-	-	1	-	-	-	3
*Hand/Fingers*	1	-	2	3	1	-	-	-	2	5	-	-	1	-	1	3	2	-	-	-	-	-	-	-
*Pelvic Girdle (anterior)*	-	-		-	2	-	-	-	-	2	-	-	-	-	-	-	-	1	-	-	-	1	-	-
*Pelvic Girdle (posterior)*	-	-	1	-	-	-	-	-	-	1	-	-	-	-	-	1	-	-	-	-	-	-	-	-
*Groin*	-	-	3	2	-	-	-	1	1	5	-	-	-	1	-	1	-	-	1	1	-	-	-	1
*Thigh (anterior)*	-	-	2	1	2	-	-	-	2	1	2	-	1	1	-	1	-	2	-	-	-	-	-	-
*Thigh (posterior)*	-	1	2	2	2	-	1	-	1	3	4	-	1	1	-	3	1	-	-	-	-	-	1	1
*Knee*	-	1	8	2	2	1	1	2	6	9	2	-		1	-	4	5	2	-	-	1	-	1	3
*Lower Leg (posterior)*	-	-	-	-	3	-	-	-	-	3	-	-	1	-	-	-	-	1	1	-	-	-	-	-
*Ankle*	-	-	5	2	3	-	-	-	5	5	-	-	1	1	1	1	2	1	-	1	-	2	-	-
*Foot/Toes*	-	-	1	-	1	-	-	1	1	1	1	-	-	1	-	1	1	-	-	-	-	-	-	-
Injury Type																								
*Bone Injury*	1	1	4	6	2	-	-	-	4	9	1	-	1	-	1	4	5	-	1	1	-	-	-	1
*Cartilage Injury*	-	-	1	-	-	1	-	1	1	2	-	-	-	-	-	-	-	-	-	-	1	-	-	2
*Concussion*	-	1	1	-	-	-	-	-	-	2	-	1	-	-	-	-		-	1	-	-	-	-	-
*Dental Injury*	-	-	-	1	1	-	-	-	-	1	1		-	-	-	-	2	-	-	-	-	-	-	-
*Joint Injury*	-	-	6	2	3	-	-	-	3	7	1	1	1	1	1	2	1	-	1	1	-	1	-	1
*Laceration*	-	-	2	1	-	-	-	-	1	1	1	-	-	-	-	1	1	1	-	-	-	-	-	-
*Ligament Injury*	-	-	2	-	5	-	1	1	4	5	-	-	-	-	-	2	3	1	-	-	1	1	-	1
*Meniscal Injury*	-	-	2	-	-	-	-	1	1	1	1	-	1	1	-	-	-	-	-	-	-	-	-	1
*Muscle Injury*	-	1	7	3	10	-	-	2	5	13	5	-	-	3	4	4	3	4	1	1	-	1	1	1
*Nasal Injury*	-	-	3	-	-	-	-	-	1	2	-	-	-	-	-	-	3	-	-	-	-	-	-	-
*Pain*	-	-	4	3	-	-	-	-	3	4	-	-	-	-	-	-	-	1	2	3	1	-	-	-
*Tendon Injury*	-	1	3	2	2	-	1	-	4	2	3	-	-	-	-	4	2	2	-	-	-	-	-	1
**Injury** **Localization**	**Game Set Pieces**							**Clinical History**		**Return-to-Sport**											**Mean**			
	*Warm-Up*	*Set Attack*	*Set Defense*	*Offensive Transition*	*Defensive Transition*	*Penalty/Direct Free Hit*	*Unknown*	*First Time*	*Recurrence*	*<* *1 Week*		*1–2 Weeks*		*2–3 Weeks*	*1–3 Months*		*4–6 Months*		*7–12 Months*		*Function*		*Pain*	*Fear*
*Head*	2	1	2	1	-	-	-	5	1	1		3		1	-		-		1		-		-	5.5
*Face*	-	1	-	4	-	2	-	5	2	4		1		2	-		-		-		-		-	6
*Neck*	-	-	1	-	-	-	-	1	-	-		-		-	-		1		-		4		3	8
*Chest/Ribs*	-	-	-	-	1	-	-	1	-	-		1		-	-		-		-		-		-	5
*Lumbar Spine*	-	1	-	-	-	-	-	-	1	-		1		-	-		-		-		-		3	4
*Shoulder*	-	1	-	2	-	1	-	3	1	-		-		1	2		1		-		4.5		4.3	9
*Elbow*	-		-	-	-	1	-	1	-	-		1		-	-		-		-		-		-	-
*Forearm (anterior)*	-	1	-	-	-	-	-	1	-	-		-		-	1		-		-		-		-	-
*Wrist*	-	1	-	2	-	-	2	3	2	-		2		-	1		2		-		2.8		2	9
*Hand/Fingers*	1	2	3	1	-	-	-	6	1	1		-		3	2		1		-		2.7		3.5	2
*Pelvic Girdle (anterior)*	-	-	-	1	1	-	-	1	1	-		1		-	1		-		-		-		5	4.5
*Pelvic Girdle (posterior)*	-	-	-	-	1	-	-	1	-	-		-		1	-		-		-		2		3	-
*Groin*	-	-	-	3	2	-	1	2	4	1		3		2	-		-		-		6.5		5	6.8
*Thigh (anterior)*		1		4	-	-	-	3	2	1		1		2	-		1		-		1		-	6
*Thigh (posterior)*	1	1	2	1	2	-	1	8	-			2		3	3				-		5		5	4.8
*Knee*	1	-	1	6	6	-	3	12	5	2		4		2	4		5		-		4.6		3.9	5.9
*Lower Leg (posterior)*	-	1	-	1	1	-	-	2	1	-		-		2	1		-		-		2		-	5
*Ankle*	1	-	3	6	-	-	-	7	3	-		2		3	4		1		-		2.5		-	4.2
*Foot/Toes*	-	-	-	3	-	-	-	3	-	1		1		-	-		-		1		5		4	6.7
Injury Type																								
*Bone Injury*	3	3	4	3	-	-	1	12	2	1		2		4	5		2		-		2.5		4.5	5.3
*Cartilage Injury*	1	-	-	-	-	-	2	2	1	1		1		1	-		-		-		7		3.5	6
*Concussion*	1	1	-	-	-	-	-	2	-	-		1		-	-		-		1		-		-	6
*Dental Injury*	-	-	2	-	-	-	-	1	1	1		-		1	-		-		-		-		-	5
*Joint Injury*	1	1	1	6	1	1	-	8	3	-		3		3	4		-		1		4.2		3.8	6.2
*Laceration*	-	1	-	1	1	-	-	2	1	1		-		2	-		-		-		-		-	5
*Ligament Injury*	-	-	1	5	2	-	1	5	4	-		1		2	6		-		-		4.6		5	7
*Meniscal Injury*	-	-	-	2	-	-	1	1	2	1		1		-	-		1		-		4.5		4.5	4.7
*Muscle Injury*	-	4	3	7	7	1	1	16	7	3		8		8	3		1		-		2.7		6	5.2
*Nasal Injury*	-	-	-	3	-	-	-	3	-	2		-		1	-		-		-		-		-	6
*Pain*	-	1	3	2	1	-	-	6	1	-		4		1	-		1		1		3		3	5
*Tendon Injury*	-	-	1	7	-	-	1	7	2	-		2		1	1		5		-		3.9		3.8	7.1

Note: 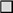 0% to ≤25%; 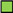 >25% to ≤50%; 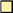 >50% to ≤75%; 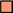 >75% to 100%.

**Table 4:** Spearman correlations between injury, sport, and sociodemographic variables (*n* = 89).

Variables	Age	Hockey Years	Hockey Injuries
Hockey years	0.826 ***	-	-
Hockey injuries	0.262 ***	0.157 *	-
1-year Hockey injuries	0.158 *	0.157 *	0.548 ***

Note: only the statistically significant correlations with injuries are displayed; *** *p* ≤ 0.001; * *p* ≤ 0.05.

**Table 5:** Logistic regressions between the sociodemographic, sport, and injury variables (*n* = 89).

Injury	Factor-Level	Odds Ratio (95% CI)	*p*	*R* ^2^
Joint	*Rink Hockey years*		*0.011*	*0.224*
	11–15	0.116 [0.019; 0.695]	0.018	
	16–19	0.116 [0.019; 0.695]	0.018	
	≥20	0.056 [0.006; 0.534]	0.012	
	≤10	Reference		

Abbreviations: CI = confidence interval; *p* = *p*-value; R^2^ = Nagelkerke. Note: only the significant statistically regressions with injuries are displayed.

## Data Availability

The data presented in this study are available on request from the corresponding author.

## Supplementary Materials

The following supporting information can be downloaded at: https://www.mdpi.com/article/10.3390/jfmk11030260/s1, Table S1: Rink Hockey practitioner’s variables by sex and age (n = 89).

## Abbreviations

The following abbreviations are used in this manuscript:

CI	Confidence Interval
h	Hour
HR	Heart Rate
kg	Kilogram
km	Kilometer
m	Meter
max	Maximum
min	Minute
mL	Milliliter
mmol	Millimole
OR	Odds Ratio
SD	Standard Deviation
