# Supplementary material for: Injury Patterns in Portuguese Under-23 and Senior Rink Hockey Athletes: A Retrospective Cross-Sectional Study
Source: J Funct Morphol Kinesiol. 2026 Jun 29;11(3):260. doi: 10.3390/jfmk11030260 (PMC13398163; doi:10.3390/jfmk11030260)
Supplement: Supplementary file 1 [file jfmk-11-00260-s001.zip › jfmk-4379084-supplementary.pdf]

Supplemental Table S1. Rink Hockey practitioner's variables by sex and age (n=89).

**Injury Localization**	**Sex**		**Age**		
	Female	*Male*	*≤ 20*	*21-25*	*≥ 26*
Head	3 (8.6%)	3 (5.6%)	3 (9.7%)	2 (5.9%)	1 (4.2%)
Face	4 (11.4%)	3 (5.6%)	3 (9.7%)	3 (8.8%)	1 (4.2%)
Neck	1 (2.9%)	0 (0.0%)	0 (0.0%)	1 (2.9%)	0 (0.0%)
Chest/Ribs	1 (2.9%)	0 (0.0%)	0 (0.0%)	1 (2.9%)	0 (0.0%)
Lumbar Spine	1 (2.9%)	0 (0.0%)	0 (0.0%)	1 (2.9%)	0 (0.0%)
Shoulder	3 (8.6%)	1 (1.9%)	2 (6.5%)	1 (2.9%)	1 (4.2%)
Elbow	0 (0.0%)	1 (1.9%)	1 (3.2%)	0 (0.0%)	0 (0.0%)
Forearm (anterior)	0 (0.0%)	1 (1.9%)	0 (0.0%)	1 (2.9%)	0 (0.0%)
Wrist	1 (2.9%)	4 (7.4%)	1 (3.2%)	3 (8.8%)	1 (4.2%)
Hand/Fingers	2 (5.7%)	5 (9.3%)	1 (3.2%)	3 (8.8%)	3 (12.5%)
Pelvic Girdle (anterior)	0 (0.0%)	2 (3.7%)	2 (6.5%)	0 (0.0%)	0 (0.0%)
Pelvic Girdle (posterior)	0 (0.0%)	1 (1.9%)	1 (3.2%)	0 (0.0%)	0 (0.0%)
Groin	2 (5.7%)	4 (7.4%)	3 (9.7%)	0 (0.0%)	3 (12.5%)
Thigh (anterior)	1 (2.9%)	4 (7.4%)	0 (0.0%)	2 (5.9%)	3 (12.5%)
Thigh (posterior)	3 (8.6%)	5 (9.3%)	2 (6.5%)	1 (2.9%)	5 (20.8%)
Knee	8 (22.9%)	9 (16.7%)	6 (19.4%)	8 (23.5%)	3 (12.5%)
Lower Leg (posterior)	0 (0.0%)	3 (5.6%)	0 (0.0%)	2 (5.9%)	1 (4.2%)
Ankle	4 (11.4%)	6 (11.1%)	4 (12.9%)	4 (11.8%)	2 (8.3%)
Foot/Toes	1 (2.9%)	2 (3.7%)	2 (6.5%)	1 (2.9%)	0 (0.0%)
**Injury Type**					
Bone Injury	4 (11.4%)	10 (18.5%)	4 (12.9%)	7 (20.6%)	3 (12.5%)
Cartilage Injury	1 (2.9%)	2 (3.7%)	3 (9.7%)	0 (0.0%)	0 (0.0%)
Concussion	1 (2.9%)	1 (1.9%)	2 (6.5%)	0 (0.0%)	0 (0.0%)
Dental Injury	0 (0.0%)	2 (3.7%)	0 (0.0%)	2 (5.9%)	0 (0.0%)
Joint Injury	4 (11.4%)	7 (13.0%)	7 (22.6%)	3 (8.8%)	1 (4.2%)
Laceration	1 (2.9%)	2 (3.7%)	2 (6.5%)	1 (2.9%)	0 (0.0%)
Ligament Injury	5 (14.3%)	4 (7.4%)	1 (3.2%)	4 (11.8%)	4 (16.7%)
Meniscal Injury	2 (5.7%)	1 (1.9%)	3 (9.7%)	0 (0.0%)	0 (0.0%)
Muscle Injury	8 (22.9%)	15 (27.8%)	5 (16.1%)	8 (23.5%)	10 (41.7%)
Nasal Injury	3 (8.6%)	0 (0.0%)	1 (3.2%)	1 (2.9%)	1 (4.2%)
Pain	4 (11.4%)	3 (5.6%)	2 (6.5%)	4 (11.8%)	1 (4.2%)
Tendon Injury	2 (5.7%)	7 (13.0%)	1 (3.2%)	4 (11.8%)	4 (16.7%)
**Injury occurrence situation**					
Warm-up (training)	0 (0.0%)	1 (1.9%)	0 (0.0%)	1 (2.9%)	0 (0.0%)
During training	18 (51.4%)	17 (31.5%)	15 (48.4%)	12 (35.3%)	8 (33.3%)
Cool-down (training)	1 (2.9%)	0 (0.0%)	1 (3.2%)	0 (0.0%)	0 (0.0%)
Warm-up (competition)	1 (2.9%)	3 (5.6%)	2 (6.5%)	2 (5.9%)	0 (0.0%)
During competition (1st half)	4 (11.4%)	14 (25.9%)	5 (16.1%)	10 (29.4%)	3 (12.5%)
During competition (2nd half)	8 (22.9%)	15 (27.8%)	5 (16.1%)	7 (20.6%)	11 (45.8%)
Cool-down (competition)	1 (2.9%)	1 (1.9%)	0 (0.0%)	0 (0.0%)	2 (8.3%)
Other	2 (5.7%)	3 (5.6%)	3 (9.7%)	2 (5.9%)	0 (0.0%)
**Injury game set pieces situations**					
Offensive transition	13 (37.1%)	23 (42.6%)	15 (48.4%)	10 (29.4%)	11 (45.8%)
Set defense	8 (22.9%)	7 (13.0%)	1 (3.2%)	8 (23.5%)	6 (25.0%)
Defensive transition	5 (14.3%)	7 (13.0%)	3 (9.7%)	5 (14.7%)	4 (16.7%)
Set attack	4 (11.4%)	7 (13.0%)	3 (9.7%)	6 (17.6%)	2 (8.3%)
Warm-up	0 (0.0%)	6 (11.1%)	3 (9.7%)	3 (8.8%)	0 (0.0%)
Penalty/Direct Free Hit	1 (2.9%)	1 (1.9%)	2 (6.5%)	0 (0.0%)	0 (0.0%)

Unknown	4 (11.4%)	3 (5.6%)	4 (12.9%)	2 (5.9%)	1 (4.2%)
**Injury in-season**					
Early-season	15 (42.9%)	12 (22.2%)	10 (32.3%)	10 (29.4%)	7 (29.2%)
Mid-season	13 (37.1%)	36 (66.7%)	18 (58.1%)	21 (61.8%)	10 (41.7%)
End-season	7 (20.0%)	6 (11.1%)	3 (9.7%)	3 (8.8%)	7 (29.2%)
**Perceived injury reason**					
Contact with another player	11 (31.4%)	13 (24.1%)	6 (19.4%)	11 (32.4%)	7 (29.2%)
Overuse	4 (11.4%)	16 (29.6%)	6 (19.4%)	7 (20.6%)	7 (29.2%)
Contact with ball	7 (20.0%)	5 (9.3%)	4 (12.9%)	5 (14.7%)	3 (12.5%)
Fatigue	2 (5.7%)	3 (5.6%)	0 (0.0%)	4 (11.8%)	1 (4.2%)
Incorrect technical gesture – stopping	2 (5.7%)	3 (5.6%)	3 (9.7%)	0 (0.0%)	2 (8.3%)
Contact with infrastructure	0 (0.0%)	3 (5.6%)	1 (3.2%)	1 (2.9%)	1 (4.2%)
Fall	1 (2.9%)	2 (3.7%)	0 (0.0%)	1 (2.9%)	2 (8.3%)
Protective equipment misplacement	1 (2.9%)	1 (1.9%)	1 (3.2%)	0 (0.0%)	1 (4.2%)
Incorrect technical gesture – spinning	1 (2.9%)	1 (1.9%)	2 (6.5%)	0 (0.0%)	0 (0.0%)
Contact with stick	0 (0.0%)	2 (3.7%)	1 (3.2%)	1 (2.9%)	0 (0.0%)
Inadequate warm-up	1 (2.9%)	0 (0.0%)	1 (3.2%)	0 (0.0%)	0 (0.0%)
Incorrect technical gesture – shooting	0 (0.0%)	1 (1.9%)	1 (3.2%)	0 (0.0%)	0 (0.0%)
Incorrect technical gesture – passing	1 (2.9%)	0 (0.0%)	0 (0.0%)	1 (2.9%)	0 (0.0%)
Unknown	4 (11.4%)	4 (7.4%)	5 (16.1%)	3 (8.8%)	0 (0.0%)
**Rink Hockey court injuries occurrences**					
1	0 (0.0%)	2 (3.7%)	2 (6.5%)	0 (0.0%)	0 (0.0%)
2	0 (0.0%)	6 (11.1%)	2 (6.5%)	0 (0.0%)	4 (16.7%)
3	2 (5.7%)	4 (7.4%)	2 (6.5%)	2 (5.9%)	2 (8.3%)
4	1 (2.9%)	1 (1.9%)	1 (3.2%)	0 (0.0%)	1 (4.2%)
5	4 (11.4%)	14 (25.9%)	9 (29.0%)	6 (17.6%)	3 (12.5%)
6	11 (31.4%)	11 (20.4%)	5 (16.1%)	11 (32.4%)	6 (25.0%)
7	5 (14.3%)	6 (11.1%)	2 (6.5%)	7 (20.6%)	2 (8.3%)
8	3 (8.6%)	2 (3.7%)	1 (3.2%)	3 (8.8%)	1 (4.2%)
9	1 (2.9%)	2 (3.7%)	0 (0.0%)	1 (2.9%)	2 (8.3%)
10	1 (2.9%)	1 (1.9%)	1 (3.2%)	0 (0.0%)	1 (4.2%)
11	2 (5.7%)	1 (1.9%)	2 (6.5%)	1 (2.9%)	0 (0.0%)
12	1 (2.9%)	0 (0.0%)	0 (0.0%)	0 (0.0%)	1 (4.2%)
Unknown	4 (11.4%)	4 (7.4%)	4 (12.9%)	3 (8.8%)	1 (4.2%)
**Return-to-sport duration**					
< 1 week	7 (20.0%)	4 (7.4%)	4 (12.9%)	3 (8.8%)	4 (16.7%)
1-2 weeks	9 (25.7%)	14 (25.9%)	10 (32.3%)	8 (23.5%)	5 (20.8%)
2-3 weeks	4 (11.4%)	18 (33.3%)	7 (22.6%)	10 (29.4%)	5 (20.8%)
1-3 months	6 (17.1%)	13 (24.1%)	6 (19.4%)	7 (20.6%)	6 (25.0%)
4-6 months	7 (20.0%)	5 (9.3%)	2 (6.5%)	6 (17.6%)	4 (16.7%)
7-12 months	2 (5.7%)	0 (0.0%)	2 (6.5%)	0 (0.0%)	0 (0.0%)
**Injury history**					
First time	30 (85.7%)	35 (64.8%)	24 (77.4%)	21 (61.8%)	20 (83.3%)
Recurrence	5 (14.3%)	19 (35.2%)	7 (22.6%)	13 (38.2%)	4 (16.7%)
**Persistence of (mean ± SD)**					
Functional limitations	4.42 (±1.32)	3.44 (±1.80)	4.38 (±1.92)	3.33 (±1.68)	4.33 (±1.63)
Pain	3.85 (±1.61)	4.00 (±1.41)	3.80 (±1.03)	3.83 (±1.85)	4.00 (±2.00)
Fear-of-injury	6.18 (±2.32)	5.56 (±2.37)	5.46 (±2.47)	6.29 (±2.44)	6.17 (±2.17)
